# SARS-CoV-2 and Influenza Vaccines in People with Excessive Body Mass—A Narrative Review

**DOI:** 10.3390/life12101617

**Published:** 2022-10-17

**Authors:** Julia Drożdżyńska, Wiktoria Jakubowska, Marika Kemuś, Martyna Krokowska, Konrad Karpezo, Marcelina Wiśniewska, Paweł Bogdański, Damian Skrypnik

**Affiliations:** 1 Faculty of Medicine, Poznan University of Medical Sciences, Fredry St. 10, 61-701 Poznan, Poland; 2 Department of Treatment of Obesity, Metabolic Disorders and Clinical Dietetics, Poznan University of Medical Sciences, Szamarzewskiego St. 82/84, 60-569 Poznan, Poland

**Keywords:** SARS-CoV-2, influenza, vaccine, antibody titre

## Abstract

In the face of a growing number of overweight people and two widely known viral diseases, SARS-CoV-2 and influenza, it is crucial to be aware of the impact of excess body weight on immunisation against these diseases. The aim of this review is to show the effectiveness of SARS-CoV-2 and influenza vaccines in overweight and obese patients. Excessive adipose tissue releases cytokines and maintains local hypoxia, which causes persistent low-grade inflammation. These factors make excess body mass patients’ immune systems weaker. Under such conditions, the humoral response becomes less efficient, leading to a weakened ability to fight against infection and an increased risk of developing lower antibody titres. Vaccines help to reduce morbidity both in normal-weight and excess body mass people, although most studies show that patients with higher BMI tend to lose the antibodies produced more quickly. It is shown that the most effective vaccines (in terms of preventing the infection and potential post-illness complications) are the BNT162b2 vaccine against SARS-CoV-2 and the inactivated influenza vaccine against influenza among both obese and non-obese subjects.

## 1. Introduction

Obesity is a worldwide condition that is spreading among humans at an alarming rate. The latest accurate survey conducted by the WHO (World Health Organisation) stated that more than 650 million people [1] were obese and the newest prognoses predict ⅓ of the population to be overweight or obese by 2025. This is a serious medical problem leading to a range of health-threatening consequences, e.g., hypertension, chronic low-grade inflammation, diabetes, cancer genesis, and an altered immunological response [2]. The costs of treatment of obesity are about to reach PLN 1073 billion (10^12^) in 2025 [3]. During the SARS-CoV-2 pandemic, obesity showed another face: inadequate or incorrect responses to vaccination in obese subjects. According to the WHO, as of 13 October 2022, SARS-CoV-2 has caused over 612 million cases of SARS-CoV-2 including more than 6.5 million deaths [4]. These are large numbers that we are still trying to overcome.

Another ongoing viral disease is influenza, which had a major outbreak in 1918 [5]. Since then, the virus has changed its nucleotide sequence to adapt to vaccinated organisms and changing conditions [6]. According to the WHO, each year, there are over 10^9^ cases of influenza noted globally and 290,000–650,000 influenza-related (respiratory complications) deaths [7]. When it comes to healthcare statistics, having excessive body fat doubles the risk of suffering from influenza [8].

In order to reduce morbidity due to infectious diseases, global scientific consensus indicates that the majority of the population should be vaccinated. Thanks to vaccine programs, it is possible to significantly prevent the spreading of those diseases. Studies show that, over the years, vaccines against influenza were able to reduce morbidity by 40–60% depending on the vaccine type. Overall, it is said that the vaccine is most effective among children, as vaccine effectiveness (VE) reaches 64% [9]. In the general settlement, it accounts for 31% lower risk of death from influenza [10]. Statistics regarding vaccination against influenza are accurate due to the prolonged period of using these vaccines.

A study conducted in the US reported that the SARS-CoV-2 vaccine markedly reduces morbidity in the elderly (≥65 years old) by 54–62% and reduces the overall death rate by 69.3%, resulting in lowering costs of treatment [11]. Currently, SARS-CoV-2 vaccines are estimated to have overall 95% efficacy in both normal-weight and obese patients (Pfizer [New York, NY, USA]—BioNTech [Mainz, Germany] and Moderna [Cambridge MA, USA]). The information regarding the SARS-CoV-2 vaccine (4 February 2022) given by the Office for National Statistics in the United Kingdom states that individuals after their booster dose are 93.4% less likely to die [12]. 

Given that patients with elevated body mass have a greater risk of suffering from influenza and SARS-CoV-2 and are more prone to developing severe disease due to a decreased response to pathogens, healthcare professionals and the whole population should be aware of the importance of vaccines.

The aim of this review was to present the newest studies regarding the effect of SARS-CoV-2 and influenza vaccines in individuals with excessive body mass. The authors’ purpose was to present the abnormalities in the immune response to those vaccines in excess body mass patients in the context of immune system disorders caused by overweight and obesity and to summarise studies on the efficiency of SARS-CoV-2 and influenza vaccines in excess body mass individuals. 

Moreover, as far as the authors are aware, there has been no single study investigating and comparing, in a single trial, immune responses to both SARS-CoV-2 and influenza vaccines, especially in individuals with excess body mass. 

## 2. Methods and Materials

**Figure 1:**
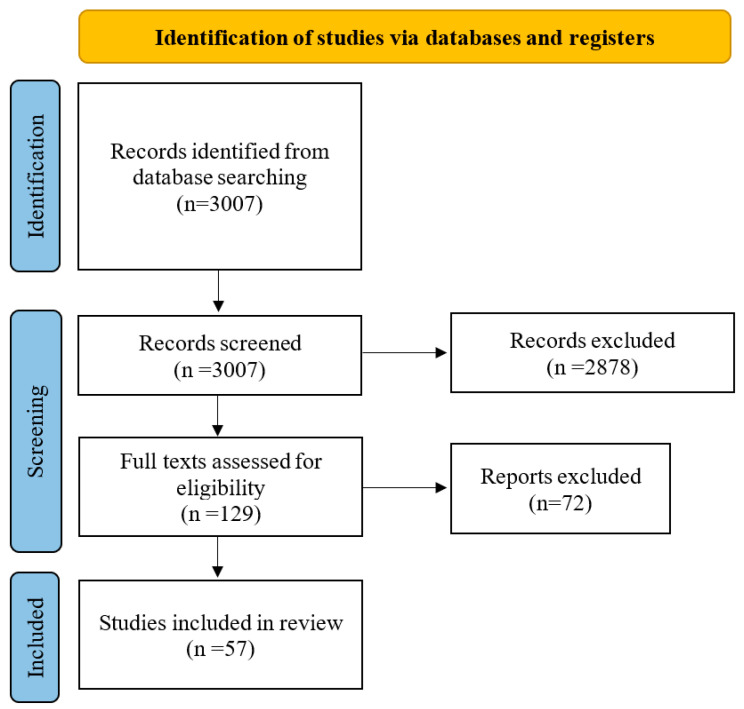
The study flow diagram.

Articles examining the association between excess body mass and immune response, excess body mass and SARS-CoV-2vaccination, and excess body mass and influenza vaccines were searched. Based on the Preferred Reporting Items for Systematic Reviews and Meta-Analyses (PRISMA) guidelines, PubMed, Web of Science, and Google Scholar were searched to identify the appropriate sources using the English language as a restriction. In total, 3007 articles were found. After reviewing the abstracts, 2878 were excluded as unrelated to the subject of our paper. The full texts of the remaining 129 articles were analysed, and a total of 57 studies were finally included in this narrative review. Keywords used were: ‘SARS-CoV-2’ or ‘coronavirus’ or ‘influenza vaccine’ and ‘obesity’ or ‘overweight’ or ‘abdominal fat’ or ‘abdominal obesity’ or ‘metabolic disturbance’ or ‘central obesity’ or ‘high BMI’ and ‘immune system’ or ‘immune response’ individually or in combination. All the included works met the following criteria: original research articles written in English and dealing with excess body mass and SARS-CoV-2 or influenza vaccination and published in 2012 or later. Review papers or papers published before 2012 were only included when the crucial information could not be found in any other available resources. Non-original research articles, duplicated articles, or publications in languages other than English were excluded. The study flow diagram is presented in Figure 1.

## 3. Results

### 3.1. Immune Response in Excess Body Mass Patients

The increasing problem of obesity, which has turned into a pandemic, especially in developed countries, has raised the question: how does excess body mass affect the immune response in these patients? Obesity is associated with low-grade chronic inflammation [13,14,15,16,17,18,19,20]. Inflammation in obesity is mainly caused by the overproduction of pro-inflammatory cytokines in adipose tissue. Expansion in macrophages was observed with increasing serum concentration of inflammatory molecules tumour necrosis factor-alpha (TNF-α) and interleukin 6 (IL-6) [13]. Elevated concentrations of some inflammatory cytokines are seen in obesity, such as interleukin 4 (IL-4), monocyte chemoattractant protein 1 (MCP-1), IL-6, and TNF-α [14]. The leukocyte and monocyte counts in peripheral blood are increased in obese patients. Circulating peripheral blood mononuclear cells (PBMC) secrete higher amounts of TNF-α in obese individuals and lower amounts of IL-10, which has an anti-inflammatory effect [13]. On the other hand, decreased secretion of interferon type I (IFN), which is an important part of the antiviral immune response, may be observed in obesity [16]. 

**Figure 2:**
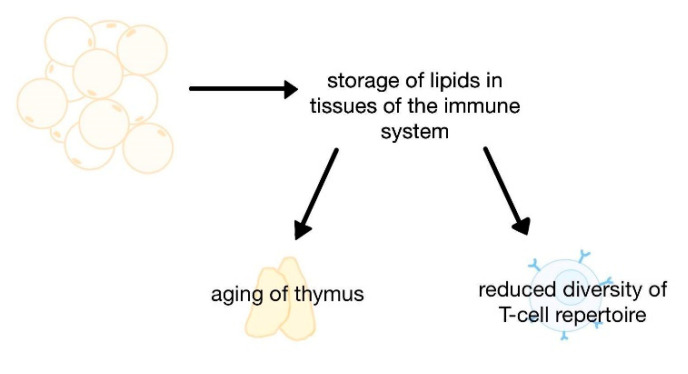
The influence of obesity on the storage of lipids in the thymus.

As the disease progresses, the activity of adipose tissue and adipocytes changes [13]. Adipocyte hypertrophy, which is predominant in adults [21], and the associated loss of the ability to accumulate fat per se, lead to inflammation by increased infiltration and polarisation of immune cells towards fatty tissue macrophages. Lipid migration to organs without adipose tissue and ectopic accumulation of fat leads to lipotoxic effects and dysfunction of many organs [14]. Obesity increases the storage of lipids in the tissues of the immune system, including the primary lymphoid organs, i.e., the thymus and bone marrow [21]. This process accelerates the aging of the thymus and reduces the diversity of the T-cell repertoire [21,22]. Studies in C57BL/6 mice have demonstrated that obesity induced by a high-fat diet causes changes in the structure of the thymus. These changes are like the process of involution that occurs with age, including loss of the corticospinal tract, increased perithymic adiposity, and a decline in the population of precursor lymphocytes. In addition, the process of gaining immunocompetence by these lymphocytes is disturbed [21], T-cell receptor (TCR) diversification is limited, and metabolic reprogramming of effector CD8+ T cells is impaired. The influence of obesity on the storage of lipids in the thymus is presented in Figure 2.

Obesity has also been shown to have an unfavourable effect on the functioning of secondary lymphoid tissues. Studies have shown that, in the spleen of mice, a high-fat diet was associated with a greater number of effector/memory cells compared to the control group. These processes decrease the number of circulating T cells, which reduces the range of pathogenic antigens to which they can respond [21]. The presence of adipocytes in the bone marrow inhibits haematopoiesis [23]. Studies in C57BL/6 mice have shown that obesity caused by a high-fat diet alters the proportions of the leukocyte progenitor cell population, with a decrease in the number of lymphoid progenitor cells. It has also been demonstrated that obesity correlates with reduced size of the inguinal lymph nodes and impaired lymphatic transport as well as the migration of dendritic cells to peripheral lymph nodes [21]. 

Adipocytes produce adipokines that play an immunomodulatory role [13], including pro-inflammatory leptin and anti-inflammatory adiponectin. Obesity leads to an increase in the level of leptin and resistin and a decrease in the level of adiponectin in the serum. This relationship between adipokines contributes to systemic inflammation [15]. In obesity, we observe hyperleptinaemia, which occurs simultaneously with central resistance to its anorexigenic effects. Leptin increases the release of pro-inflammatory cytokines and phagocytosis by monocytes, dendritic cells, and macrophages. Moreover, leptin increases neutrophil migration, cytotoxic activity, and INF-γ secretion in NK cells; however, prolonged exposure to leptin reduces proliferation and NK cell function. This specific condition related to obesity decreases the number of NK cells and their cytotoxicity. IL-6 also contributes to the weakening of the cytotoxic properties of NK cells, and leptin induces the reprogramming of T cells that promotes the polarisation of Th1 and Th17 cells [14]. Adiponectin has been shown to alter the cytotoxic effect of NK cells [19]. Elevated leptin and reduced adiponectin levels in combination with increased expression of MHC-II adipocytes leads to pro-inflammatory Th1 differentiation [24]. Decreased regulatory T-cell levels are also observed in obesity [13].

Excessive adipose tissue may result in hypoxia of the microenvironment and triggers a local inflammatory reaction due to adipocyte necrosis [16]. The released chemotactic pro-inflammatory mediators may lead to leukocyte infiltration [16].

In hypertrophic adipose tissue, there is a higher percentage of macrophages compared to healthy adipose tissue. Their level rises from 5 to 10% to even 50% [21]. The results of a human study have shown that, in visceral and perivascular adipose tissue, macrophage infiltration is twice as high as in subcutaneous adipose tissue. Infiltrated macrophages in adipose tissue differentiate from the anti-inflammatory M2 phenotype to the pro-inflammatory M1 phenotype. Saturated fatty acids in which adipose tissue is abundant activate these macrophages [14].

Obesity and related systemic inflammation impair the immune system, making it more susceptible to infection and less responsive to vaccination [14,15,16,20]. There is evidence that obesity can cause a weakening of the immune response to vaccination compared to normal-weight subjects.

The issue of vaccine effectiveness in excess body mass individuals was firstly investigated in relation to the hepatitis B vaccine [22]. Further studies on the association between obesity and response to tetanus [25] and rabies vaccines showed lower or inadequate antibody titres in obese subjects compared to normal-weight subjects [22].

**Figure 3:**
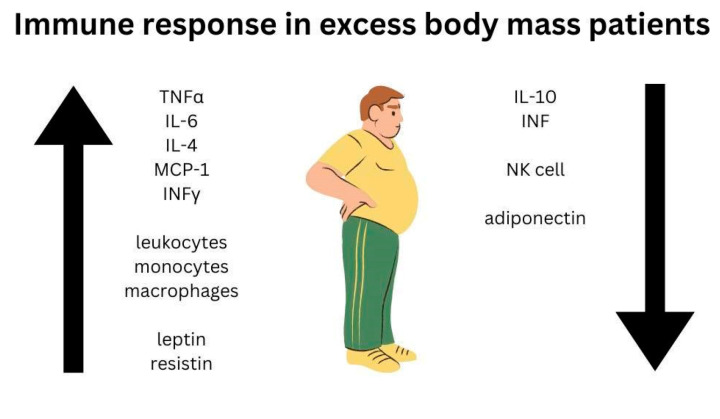
Immune response in excess body mass patients summary; IFN: interferon; IFNγ: interferon-gamma; IL-10: interleukin-10; IL-4: interleukin-4; IL-6: interleukin-6; MCP-1: monocyte chemoattractant protein-1; NK cells: natural killer cells; TNFα: tumour necrosis factor-alpha.

It has been shown that an impaired immune response to vaccination in excess body mass subjects may be due to the impaired production of plasma cells that produce antibodies. One reason for this may be, among others, reduced absorption at the injection site. Studies have demonstrated that the use of longer needles results in higher levels of antibodies against the surface antigen of the hepatitis B virus in obese adolescents [19]. Immune response in excess body mass patients is presented in Figure 3.

### 3.2. Obesity and SARS-CoV-2 Vaccination

COVID-19 is a contagious respiratory illness. It is caused by SARS-CoV-2 [26], a single-stranded RNA virus. It is built of membrane glycoproteins (M), spike proteins (S), hemagglutinin ester dimer proteins (HE), nucleocapsid proteins (N), and envelope proteins (E). The primary target of vaccines are S-proteins located on the surface of the virus [27]. There are different types of vaccines against SARS-CoV-2, e.g., mRNA vaccines (BNT162b2 and mRNA-1273), adenoviral-vectored vaccines (ChAdOx1, Gam-COVID-Vac, Ad26.COV2.S), protein subunit vaccines (NVX-CoV2372), and whole-cell inactivated viral vaccines (BBV152) [28].

One of the studies [29] performed on 252 healthcare workers at the Istituti Fisioterapici Ospitalieri (Rome, Italy) showed higher antibody levels after the first dose of BNT162b2 vaccination in the underweight and normal-weight groups vs. the pre-obesity group. The highest antibody GMC (geometric mean concentration) was noticed among underweight patients (GMC = 70.21 AU/mL) and lower among normal-weight (GMC = 58.44 AU/mL), pre-obese (GMC = 40.39 AU/mL), and obese (GMC = 39.26 AU/mL) patients (*p* < 0.0001) [28]. 

Similar results were noticed by the same group of scientists [30] who analysed the number of antibodies after the second dose of the BNT162b2 vaccine. The study was performed on 248 healthcare workers. An increased amount of antibody titres was noticed among underweight (GMC = 455.41 AU/mL) and normal-weight (GMC = 325.84 AU/mL) people in comparison to pre-obese (GMC = 222.40 AU/mL) and obese (GMC = 167.05 AU/mL) patients (*p* < 0.0001).

Another study [31], which showed a difference in the immunological response to COVID-19 vaccination among people with different BMI, was conducted at the Centre of High Specialisation for the Cure of Obesity (CASCO), Polyclinic Umberto I, Rome. The results indicated that weight loss of obese people increases immunological response to anti-COVID-19 mRNA vaccination. The subjects underwent two anti-COVID-19 vaccinations, one before BMI reduction and one afterwards. The median BMI of the subjects (all female) before BMI reduction was 40.95 kg/m^2^. Scientists noticed a correlation between cell-mediated and humoral immunological response and increased BMI (IFNγ1: R = 0.558; *p* = 0.013; IFNγ-2: R = 0.581; *p* = 0.009; antibodies: R = 0.512; *p* = 0.018). Later, the subjects’ BMI (median = 36.83 kg/m^2^) was reduced and after 21 days their immunological response to the second dose of vaccine was measured again. Weight loss did not affect IFNγ-1 and antibodies concentration but there was a correlation between weight loss and IFNγ-2 concentration. Subjects after weight loss had a higher level of IFNγ-2 (R = 0.471; *p* = 0.042) [30].

However, a study [32] performed on 86 healthcare workers with an average BMI equal to 22.4 ± 5.5 kg/m^2^ showed that subjects with BMI ≥ 30 kg/m^2^ did not have a lower immune response to BNT162b2 vaccination. There was a correlation between increased waist circumference and lower level of anti-SARS-CoV-2 antibodies afterBNT162b2 vaccination. Subjects with increased waist circumference had lowerSARS-CoV-2 antibody titres (R = −0.324; *p* = 0.004) in comparison to those with normal waist circumference [32].

On the contrary, The Obesity Society’s study [33] gave different results. The authors compared studies performed by the producers of BNT162b2, mRNA-1273, Ad26.COV2.S, and ChAdOx1-S vaccines. The BNT162b2 vaccine was efficient in 95.4% (95% CI: 86.0–99.1%) among obese subjects and 94.8% (95% CI: 87.4–98.3%) among those without obesity, which is not a remarkable difference. It was also noticed that obese individuals aged 16–64 have similarly efficient response (94.9%; 95% CI: 84.4–99.0%) as those aged above 65 years old (100.0%; 95% CI: 27.1–100.0%). Another result was that there was no significant difference in response between obese and non-obese people vaccinated with mRNA-1273. The vaccine was efficient in 94.1% (95% CI: 89.3–96.8%) among all the subjects (obese and non-obese), 95.8% (95% CI: 82.6–99.0%) for those with BMI ≥ 30 kg/m^2^, and 91.2% (95% CI: 32.0–98.9%) of participants with severe obesity (BMI ≥ 40 kg/m^2^) (one case of severe COVID-19 illness). The Ad26.CoV2.S vaccine was 66.8% (95% CI: 54.1–76.3%) efficient after 14 days and 65.9% (95% CI: 47.8–78.3%) after 28 days since the first dose among patients with BMI ≥ 30 kg/m^2^, while the efficiency among obese and non-obese together was 66.9% (95% CI: 59.0–73.4%) after at least 14 days and 66.1% (95% CI: 55.0–74.8%) at least 28 days after the first dose [33].

Similarly to The Obesity Society’s study [34], a study analysing the University of Miami Hospital’s Emergency Department patients showed that increased BMI is not negatively affecting immune response to mRNA vaccines: BMI < 25 kg/m²: 66.3% vaccine effectiveness calculated from the formula: 1 − [(a/b)/(c/d)] ((a) vaccinated patients who were tested positive for COVID-19, (b) vaccinated patients who were tested negative for COVID-19, (c) unvaccinated patients who were tested positive for COVID-19, (d) unvaccinated patients who were tested negative for COVID-19); BMI 25–29 kg/m^2^: 71.3%; BMI 30–34 kg/m^2^: 84.5% and BMI ≥ 35 kg/m^2^: 72.7%, *p* < 0.001. The study suggested that increased BMI can lead to an even greater response to vaccination. However, the scientists admit that the data could be influenced by the low number of vaccinated patients (108 patients) [34]. 

Another study suggesting an increased response to the vaccine among obese subjects was conducted at the Veer Surendra Sai Institute of Medical Sciences and Research in India. In this study, 122 healthcare professionals and healthcare facility employees after the first and second dose of ChAdOx1 vaccine had their antibody GMT measured. People with BMI < 25 kg/m^2^ (68 people; GMT = 100 BAU/mL one month after first dose; GMT = 171.70 BAU/mL one month after second dose; GMT = 111.32 BAU/mL six months after second dose) had lower antibody response to ChAdOx1 vaccine in comparison to those with BMI > 25 kg/m^2^ (54 people; GMT = 180.63 BAU/mL one month after first dose; GMT = 183.03 BAU/mL one month after second dose; GMT = 114.64 BAU/mL six months after second dose) [35]. A summary of the research on obesity and SARS-CoV-2 vaccination is presented in Table 1.

### 3.3. Obesity and Influenza Vaccination

Influenza, similar to COVID-19, is a contagious respiratory illness. Influenza viruses contain segmented, single-stranded RNA that is enclosed by a lipid bilayer membrane called the viral envelope and matrix protein M1. There are four types of influenza viruses: A, B, C, and D [36]. Types A and B have eight RNA segments and encode at least 10 proteins. Types C and D have seven RNA segments and encode nine proteins. However, only the first three are responsible for the occurrence of the disease in humans. Influenza A virus is divided into subtypes based on the conformation of hemagglutinin (HA) and neuraminidase (NA), which are the glycoproteins on the surface of the virus. They are accountable for the immunogenicity of the flu virus [37]. Hemagglutinin binds to sialic acid-containing proteins on the host cell in order to invade respiratory epithelial cells. NA cleaves the neuraminic acid sugar moieties of the host and helps in budding and release of progeny flu viruses from the infected cell.

Recombinant influenza vaccine (RIV), live attenuated influenza vaccine (LAIV), and inactivated influenza vaccine (IIV) are currently the available types of anti-influenza vaccines. Most of them contain structural elements of hemagglutinin in order to stimulate the immune system to elicit antibodies [38]. Annual vaccination is a recommended strategy to prevent infection for people aged ≥6 months. In fact, unvaccinated obese children developed influenza three times more often than vaccinated obese children and missed more school days during influenza season (4.3 vs. 2.8 days per 100 school days) [39]. According to the WHO [40], between September 2020 and January 2021, less than 0.2% of tests for influenza were positive, while the average in the 2017–2020 seasons was 17%. From February to August 2021, influenza A and B were the most frequent types circulating in Europe. However, influenza A was detected most frequently, with the domination H1N1.

#### 3.3.1. CD8+ Cells

The study by Sheridan [41] et al. showed that CD8+ cytotoxic T cells destroy cells that are infected by intracellular pathogens, including influenza virus. CD69 regulates the secretion of IFN-γ and the differentiation of regulatory T cells. Peripheral blood mononuclear cells (PBMCs) obtained from obese individuals are likely to have a significantly lower increase in activated CD8+ cells expressing CD69 compared to normal-weight subjects (*p* = 0.015) after contact with H1N1 live vaccine strain ex vivo [42]. However, the total number of the cells seems to be similar between obese and non-obese people. Levels of the CD8+ cells expressing IFNγ (*p* = 0.006) and granzyme B (GrB) (*p* = 0.026), which are able to limit replication of the virus [43], were reduced for obese compared to normal-weight individuals. In another study, obesity was associated with a decreased number of activated CD8+ T cells expressing CD28, CD40 ligand (CD40L), IFNγ as well as both IFNγ and GrB, and both CD28 and interleukin 12 receptor (IL12R) [42]. CD28 stabilises the immune synapse and facilitates T cells activation and survival as well as increases the upregulation of CD40L. CD40 on the surface of the dendritic cells binds CD40L, whereupon it stimulates the proliferation of CD4+ T cells. IL12R binds interleukin 12, which is able to regulate the response of T cells and natural killer cells.

Recent evidence indicates that obese adolescents (BMI ≥ 85th percentile) with unhealthy metabolic phenotype have a lower concentration of interleukin-13 (IL-13) (*p* = 0.04) as well as interleukin-10 (IL-10) (*p* = 0.07) compared to non-obese (BMI ≤ 85th percentile) and obese participants with healthy phenotype one month after vaccination [43]. Both IL-13 and IL-10 inhibit the production of pro-inflammatory cytokines. Adolescents with unhealthy metabolic phenotype were defined as subjects with two or more of the five cardiometabolic risk factors: waist circumference ≥ 90th percentile, triglyceride level ≥ 110 mg/dL, high-density lipoprotein (HDL) level ≤ 40 mg/dL, both systolic and diastolic blood pressure ≥ 90th percentile, and fasting plasma glucose ≥ 100 mg/dL. Children with a healthy phenotype manifested only one of the five risk factors [44].

#### 3.3.2. CD4+ and Dendritic Cells

CD4+ T cells produce and secrete cytokines in order to enhance the activation and function of CD8+ cytotoxic T cells. They are required for a comprehensive immune response to the influenza virus. CD80 and CD86 transmit second signals to T cells. Major histocompatibility complex class II (MHC-II) is responsible for the presentation of antigens to T cells. Interleukin 7 stimulates the development of T cells. Obese (mean BMI 37.8 ± 7.9 kg/m^2^) and overweight (mean BMI 27.2 ± 1.5 kg/m^2^) individuals showed no differences in proliferation, activation of dendritic cells, or expression of CD80, CD86, MHC II, IL-12, and IL-7 compared to healthy weight (mean BMI 22.3 ± 1.6 kg/m^2^) vaccinated subjects. However, increased BMI level does impair the activation of CD4+ T cells resulting in lower expression of CD28, CD40L, IL12R, CD69, INF-γ, and GrB (*p* < 0.05) [43].

#### 3.3.3. HAI Titres, Seroconversion and Seroprotection

The hemagglutination inhibition assay (HI test) is used to classify viruses by the inhibition of hemagglutination with subtype-specific antibodies [44]. Furthermore, it can help to evaluate the immunogenicity of influenza vaccination. Data suggests that HAI antibody titres ≥ 1:40 are correlated with protection from influenza infection, which is defined as seroprotection [45]. Seroconversion is defined as a four-fold or greater increase in HAI titre after vaccination. It is interesting to note that Sheridan et al. [41] observed higher antibody responses 1 month after vaccination in participants with elevated BMI (mean BMI 35.7 ± 4.5 kg/m^2^) than in healthy weight (mean BMI 22.2 ± 1.7 kg/m^2^) individuals for the influenza B vaccine component (*p* = 0.04). There was no difference in the response between vaccinated groups for the influenza H3N2 and influenza H1N1 vaccine components (*p* = 0.09 and *p* = 0.014, respectively). Such a lack of difference was also shown by Neidich et al. [8]. In contrast, another study discovered that obesity (BMI ≥ 30 kg/m^2^) was correlated with an increased seroconversion rate to H3N2 virus strain vaccination. Seroconversion was adding up to 73% for BMI ≥ 30 kg/m^2^ vs. 62% for BMI < 30 kg/m^2^ in a group of ≥50 year old adults [46]. Similarly to Sheridan et al., other studies found no interrelation between seroconversion, seroprotection, and elevated BMI level to different influenza H1N1 vaccine components in adults and children after two doses [47] and for H1N1, H3N2, and influenza B lineages in children [44]. Furthermore, adults aged <60 years with first-class obesity had a higher rise of HAI titres for H3N2 vaccine strain in comparison with obesity in classes two and three (*p* < 0.001) [48]. Pregnant and postpartum obese women showed lower seroconversion rate than non-obese women (51.5% and 64.4%, respectively [49]).

Twelve months after vaccination, increased BMI resulted in a greater decrease in HAI vaccination titres. More than 50% of obese participants had a four-fold or larger decrease in seroconversion for influenza B and 47% for H1N1 vaccine strain, while it was observed only in <25% normal-weight participants [42,50].

#### 3.3.4. Risk of Developing Influenza and Influenza-like Illness

It was proposed that a higher HAI titre does not correlate with protection from influenza or influenza-like illness (ILI). ILI is defined by the WHO as a respiratory infection with onset within the last 10 days, an occurrence of cough, and fever ≥ 38 °C [51]. Vaccinated obese individuals were twice as likely to suffer from confirmed influenza or ILI than vaccinated normal-weight subjects (relative risk = 2.01, 95% CI 1.12, 3.60, *p* = 0.020). Opposingly to this study, Smit et al. observed that obese vaccinated children had a similar incidence of influenza or ILI as non-obese children (61.8 vs. 54.7 per 1000 children, *p* = 0.418). However, obese children missed more school days during influenza season than children with healthy weight (4.3 days vs 3.8 days per 100 school days, *p* = 0.002). A summary of the research performed on obesity and influenza vaccination is presented in Table 2.

### 3.4. Additional Comorbidities

Being overweight increases the risk of developing hypertension, type 2 diabetes, and various heart diseases. In addition, due to immune impairment, a patient can bear a disease badly and suffer from other conditions weakening the immune response, which implies that the antibody titre may differ.

Taking into consideration patients with common variable immunodeficiency (CVID) and their SARS-CoV-2 vaccination response, all of the patients have developed a humoral response [52]. However, 24% of CVID patients did not produce antigen-specific T cells required for complete immunity. The situation with influenza vaccine responses is completely opposite. According to another study, only 16.7% of CVID patients produced the required antibodies [53]. Therefore, it can be supposed that the immune response of obese patients may differ not only due to obesity, but also due to other concomitant diseases.

Another immune-related disease that modifies antibody production is rheumatoid arthritis, a condition frequently acquired by overweight patients. In this case, both the BNT162b2 vaccine and the trivalent influenza vaccine turned out to be effective in creating a humoral response in rheumatoid arthritis patients. Respectively, 77% [54] and 81.2% [55] of the study participants developed humoral responses.

Unexpectedly, studies show that in patients with human immunodeficiency virus (HIV) infection, SARS-CoV-2 and influenza vaccines are very efficient. Despite HIV being CD4-specific, in the clinical trials, all vaccinated patients produced antibodies against SARS-CoV-2 [56] and influenza viruses [57]. Nevertheless, the antibody titre in the influenza vaccine trial was dropping faster when a patient was HIV-positive.

## 4. Discussion

### 4.1. Summary of Results

#### 4.1.1. SARS-CoV-2

Because of the negative effect on the immune system induced by obesity, it seems to be questionable if the vaccine against SARS-CoV-2 in obese subjects is as effective as it is in non-obese individuals. The studies show different responses to anti-COVID-19 vaccines (both more effective and less effective) among people with increased BMI. The immunological response seems to be negatively affected by BMI only for mRNA vaccines. Studies suggest that the BNT162b2 vaccine can cause a slightly lower immunological response among obese patients, while the response to the mRNA-1273 vaccine can be lower in subjects with BMI ≥ 40 kg/m^2^. Adenoviral-vectored anti-COVID-19 vaccines seem to be equally efficient or more efficient among obese subjects in comparison to non-obese subjects. However, most studies on adenoviral-vectored vaccines have been based on small groups of subjects, so their results may not be fully relevant. Due to the increasing number of obese people, more studies with a higher number of subjects are needed to properly analyse the issue of SARS-CoV-2 vaccination efficiency in excess body mass patients.

#### 4.1.2. Influenza

Obesity may constitute an independent risk of morbidity and mortality due to influenza infection. Annual vaccination is an advised approach to avoid the disease. In presented studies, the most frequently administered vaccine was inactivated trivalent influenza vaccine (TIV) [42,43,46,48,50] which protects from an influenza A (H1N1) virus, an influenza A (H3N2) virus, and one of the influenza B viruses, i.e., B/Yamagata or B/Victoria. Nowadays, the quadrivalent vaccine is the most recommended vaccine, containing, in addition, one more influenza B lineage virus according to the Advisory Committee on Immunisation Practices (ACIP).

Regardless of complete vaccination, obesity may be associated with an altered and reduced ability to maintain long-term antibody response. It is suggested that dysregulated cytokine production occurs due to chronic inflammation in obese subjects. Malfunction of T cells potentially leads to lowered immune efficiency, making the immune system more susceptible to infection and less responsive to vaccination. CD80 and CD86 transmit additional or second signals to T cells by attaching to CD28. CD28 is responsible for delivering a strong costimulatory signal in order to stimulate T cells. CD40L and CD69 stimulate the proliferation of CD4+ T cells. IL12R on the surface of T cells binds IL-12, which is able to regulate the response of T cells and natural killer cells. Therefore, significantly decreased expression of CD28, CD40L, IL12R, and CD69 in obese subjects leads to markedly lower activation of CD4+ and CD8+ T cells after influenza vaccination [42,43]. Additionally, lowered secretion of INF-γ may result in ineffectiveness of anti-viral function, and decreased levels of GrB lead to the defect of inducing the apoptosis of infected cells. However, activation of dendritic cells in obese subjects seems to be within the normal range [43].

Obese vaccinated adults developed influenza and ILI two times more frequently than healthy weight vaccinated individuals [46]. Obese vaccinated children were not more susceptible to influenza and had a similar incidence of the ILI occurrence to normal-weight children but suffered increased symptoms upon infection.

It was also hypothesised that inferior protection of obese people after influenza vaccination may be due to lower seroconversion and seroprotection rates. Nevertheless, some studies indicate that people with elevated BMI have higher rates of seroconversion [47], while other trials have indicated that there is no significant difference between the obese and non-obese groups in seroprotection and seroconversion rates [44,46,48]. One month after vaccination, HAI titres were higher in the group of obese people [41]. However, 12 months after vaccination, there was a correlation between elevated BMI level and a greater drop in HAI titres [42,51]. Serologic responses to influenza vaccination in obese adults are still not clear and need further investigation.

### 4.2. Study Limitations

This review includes various studies, but these studies differ in the quality and the quantity of the obtained data. Most importantly, the studies included subjects differing in age, number of participants, inhabited environment, and region of the world. Moreover, the studies varied in terms of the timeframe of the conducted analyses, which is important in the spread of viral diseases. Furthermore, regions of the world in which particular studies were conducted have different percentages of vaccinated people, which may have distorted the general research outcomes. Moreover, differences in the number of tests for influenza and SARS-CoV-2 performed in regions where the studies were conducted could affect the obtained data. Finally, SARS-CoV-2 vaccination was introduced too recently to fully investigate the long-term immune response to it, especially in the population of patients with obesity.

### 4.3. Study Strengths

The main strong aspect of this review is that, for the first time, the comparison of the immunological response in people with excessive body mass after receiving vaccines against SARS-CoV-2 and influenza is presented. This study included many clinical trials that provide precise statistics on vaccination and other epidemiological data. The latest research from around the world was used. In addition, details regarding the molecular context and information based on clinical trials have been explored. The authors responded to the ongoing problem and indicated particular issues, so that this knowledge can be used to understand the immunological response to vaccines in overweight and obese patients.

## 5. Conclusions

Accumulated studies have shown that excess body weight adversely affects the prognosis of a cure for influenza and SARS-CoV-2. The studies also show a positive correlation between both SARS-CoV-2 and influenza vaccinations and lower risk of post-disease complications as well as faster recovery from the discussed infections. Obese people in most studies have shown a lower immune response to the above vaccines than those with a normal BMI. Obese subjects have a lower number of activated CD4+ and CD8+ cells with decreased expression of IFN-γ, GrB, CD28, CD40L, and IL12R compared to non-obese individuals.

Obesity may be associated with lower antibody levels after SARS-CoV-2 and influenza vaccinations in comparison to non-obese subjects, which suggests a lower humoral response to vaccines. It appears that mRNA SARS-CoV-2 (BNT162b2) vaccines are more efficient among both obese and non-obese subjects than adenoviral-vectored vaccines. The inactivated influenza vaccine against influenza is the most effective type of vaccine both for obese and normal-weight patients. However, the scientific issue of the effect of COVID-19 and influenza vaccines on excess body mass patients needs further investigation to draw more precise conclusions.

## Figures and Tables

**Table 1:** Summary of research on obesity and SARS-CoV-2 vaccination.

Methodology	Result	References
A total of 252 healthcare workers were vaccinated with BNT162b2 vaccine. After 21 days, they had their IgG antibodies against antigens S1 and S2 of SARS-CoV-2 titres in their sera after taking their blood samples and nasopharyngeal swabs.	Pre-obese (GMC = 40.39) and obese (GMC = 39.26) subjects had lower antibody titres than normal-weight (GMC = 58.44) and underweight (GMC = 70.21) patients.	[29]
A total of 242 healthcare workers had their antibody titres analysed 7 days after the second dose of BNT162b2 vaccine. Blood samples and nasopharyngeal swabs were collected from them at the day of receiving the second dose of vaccine and 7 days later.	Pre-obese (GMC = 222.40) and obese (GMC = 167.05) subjects had lower antibody titres than normal-weight (GMC = 325.84) and underweight (GMC = 455.41) patients.	[30]
Adult subjects with BMI ≥ 35 kg/m^2^ were vaccinated with BNT162b2 vaccine and were put on a diet to reduce their BMI and were vaccinated with the second dose of BNT162b2 vaccine. Blood samples were collected after the first vaccination and after the second dose to measure Anti-SARS-CoV-2 antibody level, IFNγ-1 and IFNγ-2 level, and T-cell reactivity and compare them before and after weight loss.	After weight loss, the subjects had a higher level of IFNγ-2 (R = 0.471). There was no significant correlation between weight loss and anti-SARS-CoV-2 antibody levels, IFNγ-1 and IFNγ-2 levels, and T-cell reactivity.	[31]
A total of 86 healthcare workers were vaccinated with two doses of BNT162b2 vaccine (3 weeks between the first and the second dose) and had their venous blood drawn to measure antibody titres.	Subjects with increased waist circumference had lower SARS-CoV-2 antibody titres (R = −0.324) in comparison to those with normal waist circumference but no correlation between antibody titres and BMI was noticed.	[32]
The Obesity Society compared studies made by the producers of BNT162b2, mRNA-1273, Ad26.COV2.S, and ChAdOx1-S vaccines to compare their effectiveness among obese people.	The most efficient vaccines among obese people were BNT162b2 (94.8% efficient) and mRNA-1273 (95.8% efficient among subjects with BMI ≥ 30 kg/m^2^ and 91.2% among those with BMI ≥ 40 kg/m^2^) vaccines.	[33]
The study analysed effectiveness of mRNA vaccines among adult subjects in University of Miami Hospital’s ED. Vaccine effectiveness was calculated 1 − [(a/b)/(c/d)] (a—vaccinated patients who were tested positive for COVID-19, b—vaccinated patients who were tested negative for COVID-19, c—unvaccinated patients who were tested positive for COVID-19, d—unvaccinated patients who were tested negative for COVID-19).	Increased BMI can lead to an increased response to vaccination: BMI < 25 kg/m^2^: 66.3% efficient; BMI 25–29 kg/m^2^: 71.3%; BMI 30–34 kg/m^2^: 84.5% and BMI ≥ 35 kg/m^2^: 72.7%.	[34]
A total of 122 subjects’ blood was tested for IgG against SARS-CoV-2 level 1 month after first dose, 1 month after second dose, and 6 months after second dose of ChAdOx1 vaccine.	Subjects with BMI > 25 kg/m^2^ had higher levels of antibodies (GMT = 180.63 BAU/mL one month after first dose; GMT = 183.03 BAU/mL one month after second dose; GMT = 114.64 BAU/mL six months after second dose) in comparison to those with BMI < 25 kg/m^2^ (GMT = 100 BAU/mL one month after first dose; GMT = 171.70 BAU/mL one month after second dose; GMT = 111.32 BAU/mL six months after second dose).	[35]

BAU: binding antibody units; BMI: body mass index; ED: emergency department; GMC: geometric mean concentration; GMT: geometric mean titre; IgG: I: immunoglobulin G; IFNγ-2: interferon-gamma-2; S1/S2: spike glycoprotein.

**Table 2:** Summary of research on obesity and influenza vaccination.

Methodology	Result	References
The study reviewed BMI, a history of influenza, and vaccination status of 4260 children aged 5–13 years collected during a prospective study in the time of the 2010–2011 influenza season in Los Angeles. Children with suspected influenza and ILI had combined nose/throat swabs and were examined with polymerase-chain reaction.	Obese vaccinated children had similar incidence of influenza or ILI as non-obese children (61.8 vs. 54.7 per 1000 children, *p* = 0.418). Obese children missed more school days during influenza season than children with healthy weight (4.3 days vs 3.8 days per 100 school days, *p* = 0.002).	[39]
A total of 74 adults were vaccinated with inactivated TIV in the 2009–2010 influenza season, where serum samples were used in HAI in order to measure antibody response 1 month and 12 months after vaccination. At the baseline and 28–35 days after vaccination, PMBCs were obtained to evaluate markers of activation.	Twelve months after vaccination, increased BMI resulted in greater decrease in HAI vaccination titres. Obese participants had lower activation of CD8+ T cells compared to non-obese participants.	[41]
PBMCs of 45 adults vaccinated with TIV were obtained at the baseline and 28–35 days after vaccination to measure cytokines levels, activation of CD4+, CD8+ T-cells, and dendritic cells using flow cytometry, and cytokine secretion by cytometric bead array assays.	Numbers of activated CD8+ T cells expressing CD28, CD40 ligand (CD40L), IFN-γ as well as both IFN-γ and GrB, and both CD28 and interleukin 12 receptor (IL12R) were reduced for obese individuals. Increased BMI level does impair activation of CD4+ T cells resulting in lower expression of CD28, CD40L, IL12R, CD69, INF-γ, and GrB (*p* < 0.05).	[42]
A total of 43 children and adolescents aged 9–17 years were vaccinated with QIV during the 2017–2020 seasons and their serum samples were obtained and analysed for serotype-specific influenza antibody response 25–42 days after vaccination determined by HI assay. Authors compared cytokines and chemokines levels between MHOO, MUOO, and non-obese subjects.	Obese adolescents with unhealthy metabolic phenotype have lower concentration of IL-13 (*p* = 0.04) as well as IL-10 (*p* = 0.07) compared to non-obese and obese participants with healthy phenotype 1 month after vaccination. No interrelation was found between seroconversion, seroprotection, and elevated BMI level for H1N1, H3N2, and influenza B vaccine components.	[43]
A total of 1022 adults were vaccinated with TIV during the 2013–2015 influenza seasons; serum samples were used for assessment of serologic response 26–35 days after vaccination. Participants were asked to report influenza symptoms and the FDA-cleared Cepheid Xpert Flu assay was used to detect influenza.	Vaccinated obese individuals were twice as likely to suffer from confirmed influenza or ILI than vaccinated normal weight subjects (relative risk = 2.01, 95% CI 1.12, 3.60, *p* = 0.020). There was no significant difference in seroconversion and seroprotection between vaccinated weight groups for the influenza H3N2 and influenza H1N1 vaccine components.	
A total o f415 adults older than 50 years were vaccinated with TIV during the 2008–2009 season. Serum specimens were obtained at the baseline and 21–28 days after vaccination for HI assay, serologic response was measured, and the results were compared between different weight groups.	Seroconversion was adding up to 73% for BMI ≥ 30 kg/m^2^ vs. 62% for BMI < 30 kg/m^2^ in a group of ≥50-year-old adults. Obesity was correlated with seroconversion to H3N2 but not to H1N1 or influenza B vaccine component.	[46]
A total of 794 adults, 160 adolescents, and 178 children were vaccinated with two different monovalent, unadjuvanted split-virus H1N1 influenza vaccines. Serum samples were collected at the baseline, on day 8, and on day 21 and were used for HI assay after day 21 along with comparison of the immune response between normal weight and obese subjects.	No significant difference in seroprotection and seroconversion rates between obese and non-obese children, adolescents, and adults.	[47]
A total of 53 obese adults aged 21–69 years were vaccinated with QIV in the 2017–2018 season. HAI was utilised for the assessment of antibody response in blood 4–8 weeks after vaccination and serologic response was measured.	Adults aged <60 years with first class obesity had higher rise of HAI titres for H3N2 vaccine strain in comparison with class two and three obesity (*p* < 0.001)	[48]
A total of 239 antepartum and postpartum women received monovalent and TIV vaccine during the 2006–2009 influenza seasons. Immunologic responses were evaluated by hemagglutination inhibition methods using serum samples obtained at the baseline and 4–8 weeks after vaccination.	Pregnant and postpartum obese women showed lower seroconversion rate than non-obese women (51.5% and 64.4%, respectively).	[49]
A total of 4048 adults received the adjuvanted split virion or non-adjuvanted vaccine. Antibody response was assessed with HI methods before vaccination and during day 21, 42, and 182.	HI antibody response was similar between normal weight, overweight, and obese participants.	[50]

BMI: body mass index; CD: cluster of differentiation; HAI: hemagglutination inhibiting antibody; HI: hemagglutination inhibition; GrB: granzyme B; IFNγ: interferon-gamma; IL: interleukin; ILI: influenza-like illness; MHOO: healthy metabolic phenotypes; MUOO: unhealthy metabolic phenotypes; PMBC: peripheral blood mononuclear cells; TIV: trivalent influenza vaccine; QIV: quadrivalent influenza vaccine.

## Data Availability

This work is a review. The data used to prepare this work are available in the cited sources.

## Abbreviations

BAU	Binding antibody units
BMI	Body Mass Index
CASCO	Centre of High Specialisation for the Cure of Obesity
CD	Cluster of differentiation
CVID	Common Variable Immunodeficiency
ED	Emergency Department
GMC	Geometric mean concentration
GMT	Geometric Mean Titre
Grab	Granzyme B
HA	Hemagglutinin
HAI	Hemagglutination Inhibiting Antibody
HI	Hemagglutination Inhibition
HIV	Human Immunodeficiency Virus
HDL	High-Density Lipoprotein
IFN	Interferon
IFN	Gamma
IFNγ	Interferon-gamma
IgG	Immunoglobulin G
IIV	Inactivated Influenza Vaccine
IL-10	Interleukin-10
IL-6	Interleukin-6
IL12R	Interleukin 12 Receptor
ILI	Influenza-like Illness
LAIV	Live Attenuated Influenza Vaccine
MCP-1	Monocyte Chemoattractant protein-1
MHC-II	Major Histocompatibility Complex-II
MHOO	Healthy Metabolic Phenotypes
MUOO	Unhealthy Metabolic Phenotypes
NA	Neuraminidase
NK cells	Natural Killer Cells
PBMC	Peripheral Blood Mononuclear Cell
RIV	Recombinant Influenza Vaccine
TCR	T-cell receptor
TIV	Trivalent Influenza Vaccine
TNF-alpha	Tumour necrosis factor-alpha
QIV	Quadrivalent Influenza Vaccine
S1/S2	Spike Glycoprotein
WHO	World Health Organisation
